# Excellent Prognosis of Central Lymph Node Recurrence-Free Survival for cN0M0 Papillary Thyroid Carcinoma Patients Who Underwent Routine Prophylactic Central Node Dissection

**DOI:** 10.1007/s00268-018-4497-x

**Published:** 2018-01-25

**Authors:** Yasuhiro Ito, Akira Miyauchi, Hiroo Masuoka, Mitsuhiro Fukushima, Minoru Kihara, Akihiro Miya

**Affiliations:** Department of Surgery, Kuma Hospital, 8-2-35 Shimoyamate-dori, Chuo-ku, Kobe, Hyogo 650-0011 Japan

## Abstract

**Introduction:**

In Japan, prophylactic central node dissection (p-CND) for papillary thyroid carcinoma (PTC) has been routinely performed in many institutions, including ours (Kuma Hospital, Japan). We evaluated the recurrence to a central lymph node in patients with cN0M0 PTC who underwent routine p-CND.

**Materials and methods:**

We enrolled 4301 patients with cN0M0 PTC who underwent an initial surgery between 1987 and 2005 (median age 51 years). The postoperative follow-up periods ranged from 4 to 362 months (median 164 months). Only 15 patients underwent radioactive iodine (RAI) ablation (≥30 mCi) after total or near total thyroidectomy.

**Results:**

Of the 4301 patients with N0M0 PTC who underwent p-CND, 2548 (59%) were diagnosed as pN1a on postoperative pathological examination. To date, only 52 cases (1.2%) showed recurrence to a central lymph node. The 10-year and 20-year central node recurrence-free survival rates were excellent at 99.1 and 98.2%, respectively. On multivariate analysis, age ≥55 years, significant extrathyroid extension, tumor size >2 cm, and ≥5 pathologically confirmed central node metastases (but not the presence of central node metastasis) independently affected central node recurrence.

**Conclusions:**

Under the situation of routine p-CND, the central node recurrence-free survival of cN0M0 PTC is excellent. However, future studies, including double-arm studies from Japan, should examine whether the omission of p-CND cN0M0 PTC is appropriate without RAI ablation in consideration of various factors, including the pros and cons of p-CND.

## Introduction

Papillary thyroid carcinoma (PTC) is the most common malignancy arising from thyroid follicular cells. PTC usually shows an excellent prognosis unless it has aggressive clinicopathological features such as clinical node metastasis, distant metastasis at diagnosis, or significant extrathyroid extension (Ex) [1–3]. However, PTC frequently metastasizes to regional lymph nodes, namely nodes in the central and lateral compartments. In Japan, therefore, lymph node dissection has been extensively performed in PTC cases. At most institutions in Japan, central lymph nodes are routinely dissected, and prophylactic lateral node dissection (prophylactic modified radical neck dissection [p-MND]) is also frequently performed.

We demonstrated that the indication of p-MND should be limited because it did not improve patients’ lateral node recurrence-free survival rates—except for PTC > 3 cm with Ex [4]. However, the precise outcome of central node dissection has not been elucidated. According to the guidelines issued by the Japan Association of Endocrine Surgeons/Japanese Society of Thyroid Surgery, prophylactic central node dissection (p-CND) is accepted as a surgical strategy for cNM0 PTC [5]. It is not evident that p-CND improves patients’ prognoses, but the Japanese guidelines recognize the benefit of p-CND toward preventing the need for a second surgery for recurrence to the central compartment, which has a risk of recurrent laryngeal nerve injury and permanent hypoparathyroidism. Wound extension is not needed for p-CND and the procedure is not time-consuming; these factors also contribute to the acceptance of p-CND in the Japanese guidelines.

In contrast, guidelines established by the American Thyroid Association stated that p-CND should be considered in patients who have advanced primary tumors (T3 or T4) or clinically involved lateral neck nodes (cN1b) or if the information will be used to plan further steps in therapy, indicating that p-CND is not recommended for PTCs ≤ 4 cm that are negative for clinical node metastasis and distant metastasis [6]. The American and Japanese guidelines thus differ significantly regarding the indications for p-CND.

In this study, we investigated the central node recurrence-free survival of a large number of PTC patients without clinical node metastasis or distant metastasis (cN0M0) who underwent p-CND.

## Patients and methods

### Patients

We enrolled 4301 patients with cN0M0 PTC who underwent an initial surgery at Kuma Hospital between 1987 and 2005: 389 males and 3912 females aged 7–89 years (median 51 years). The diagnosis of cN0M0 was based on a preoperative clinical assessment. The N status was evaluated by ultrasound and, in some cases, by CT scan and MRI. The M status was evaluated mainly by chest CT scan. The postoperative follow-up periods ranged from 4 to 362 months (median 164 months). Our exclusion criteria were patients who (1) had undergone only locally palliative surgery or (2) had other malignancies arising from the thyroid such as follicular carcinoma, medullary carcinoma, anaplastic carcinoma, and malignant lymphoma. These patients were not enrolled in this study.

In the same period, 1118 patients were preoperatively diagnosed as N1b and underwent therapeutic or prophylactic CND and therapeutic MND. One hundred and forty-three patients were preoperatively diagnosed as N1a. All of them underwent therapeutic CND, and 107 of these also underwent prophylactic MND. Sixty-eight patients were diagnosed as cM1. These patients were not enrolled in the study.

At that time, if the tumor was solitary, the attending physician considered hemithyroidectomy or subtotal thyroidectomy. However, if the tumor size was large (e.g., ≥3 cm), the attending physician often, although not always, considered a total or near total thyroidectomy (residual thyroid ≤1 g). If multiple PTCs, other pathological lesions (such as follicular tumors and multinodular goiter in the contralateral lobe), or other diseases such as Graves’ disease coexisted with the primary tumor, a total thyroidectomy was often chosen. As a result, the extent of thyroidectomy was total thyroidectomy in 1897 patients, near total thyroidectomy in 69 patients, subtotal thyroidectomy in 439 patients, and limited thyroidectomy (such as hemithyroidectomy or isthmectomy) in the remaining 1896 patients. A complete CND (bilateral paratracheal and pretracheal lymph nodes) was performed in all of the patients who underwent a total, near total, or subtotal thyroidectomy. Other patients underwent ipsilateral paratracheal and pretracheal lymph node dissection or, although it was rare, a complete CND at the attending physician’s discretion. Bilateral or unilateral p-MND was also performed in 3205 (74.5%) of the patients.

### Postoperative follow-up

Postoperative scintigraphy using a small amount of radioactive iodine (≤13 mCi) was performed in 608 (32%) of the 1897 patients who underwent total thyroidectomy. Only 15 patients underwent adjuvant radioactive iodine therapy (≥30 mCi) after total or near total thyroidectomy. Distant metastasis was detected in none of the patients in our series on preoperative imaging studies and/or postoperative RAI therapy. All of the patients were followed by blood tests for factors (such as thyroid stimulating hormone, free T4, and thyroglobulin) and ultrasound once or twice per year to examine whether nodal metastasis and recurrence to the remnant thyroid (for the patients who underwent a limited thyroidectomy) had appeared. CT scan and bone scintigraphy were also used for follow-up according to the physicians’ discretion when distant recurrence was suspected due to the patient’s symptoms or, for patients who had undergone a total thyroidectomy, elevated thyroglobulin levels. We regarded cases as having PTC recurrence when recurred lesions were detected on any imaging study.

### Statistical analyses

We used the Kaplan–Meier method with log-rank tests to determine the central node recurrence-free survival rate. The multivariate analysis was performed with a Cox regression model and log-rank test. The software program StatView was used. *P* values < 0.05 were accepted as significant.

## Results

### Incidence of pathological central node metastasis for cN0M0 PTC patients

Of the 4301 patients with cN0M0 PTC who underwent prophylactic CND, 2548 (59%) were diagnosed as positive for central node metastasis on postoperative pathological examination. Table 1 shows the relationships between pathologically confirmed central node metastasis and various clinicopathological features. Patients with young age (<55 years), male gender, Ex based on gross intra-operative findings, and large tumor size were more likely to show pathologically positive lymph node metastasis.

**Table 1:** Relationships of pathological central node metastasis with clinicopathological features of 4301 cN0M0 PTC patients

Pathological central node metastasis				
Variables	No	Yes	Total	*p* value
Age, years				
≥55	843 (49%)	880 (51%)	1723	<0.0001
<55	910 (35%)	1668 (65%)	2578	
Gender				
Male	116 (30%)	273 (70%)	389	<0.0001
Female	1637 (42%)	2275 (58%)	3912	
Extrathyroid extension				
Yes	132 (32%)	284 (68%)	416	<0.0001
No	1621 (42%)	2264 (58%)	3385	
Tumor size				
>4 cm	73 (23%)	246 (77%)	318	<0.0001
3.1–4 cm	116 (26%)	323 (74%)	439	
2.1–3 cm	285 (31%)	638 (69%)	923	
1.1–2 cm	734 (44%)	933 (56%)	1667	
≤1 cm	545 (57%)	408 (43%)	953	

### Factors predicting central node recurrence in cN0M0 PTC patients

**Fig. 1:**
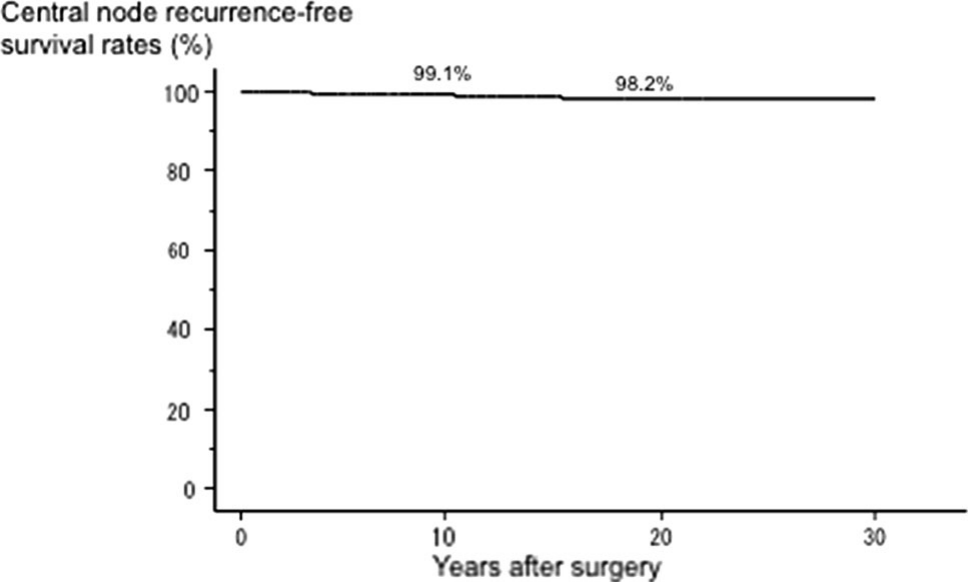
Kaplan–Meier curve of central node recurrence-free survival in 4.301 N0M0 PTC patients

To date, only 52 cases (1.2%) showed recurrence to a central lymph node. Recurrence to the nodes was based on imaging studies such as ultrasound and CT scan. In addition, we often, but not always, performed an FNAB for suspicious nodes and measured the thyroglobulin in the washout of the needles used for FNAB. The Kaplan–Meier curve analysis of the 4301 cN0M0 PTC patients for central node recurrence-free survival revealed that the 10-year and 20-year central node recurrence-free survival rates were excellent at 99.1 and 98.2%, respectively, as shown in Fig. 1.

We then investigated the prognostic value of representative prognostic factors for central node recurrence-free survival. On the univariate analysis, tumor size > 2 cm (*p* = 0.0008), tumor size >4 cm (*p* < 0.0001), Ex (*p* < 0.0001), age ≥55 years (*p* = 0.0007), and pathologically confirmed central node metastasis (*p* = 0.0159) were recognized as predictors of recurrence to the central nodes, whereas male gender was not related to central node recurrence (*p* = 0.3694). We must note that although pathological central node metastasis was more frequently observed in the patients <55 years old (Table 1), the central node recurrence-free survival of the patients ≥55 years old was poorer than that of the patients < 55 years old.

Tables 2, 3, and 4 summarize the results of the multivariate analysis. In the analysis of variables that are evaluable pre- or intra-operatively, age ≥ 55 years, Ex, tumor size measuring 2.1–4 cm, and tumor size > 4 cm independently affected central node recurrence (Table 2). We then analyzed these factors together with pathological central node metastasis, which was evaluable only postoperatively. The presence of pathologically confirmed central node metastasis was not recognized as an independent predictor of central node recurrence (Table 3). We then included the large number of pathological central node metastases (≥5) in the analysis instead of the presence of pathological central node metastasis. As shown in Table 4, number of central node metastasis ≥5 independently affected central node recurrence together with age ≥55 years, Ex, and large tumor size.

**Table 2:** Multivariate analysis of variables evaluated preoperatively or intra-operatively for central node recurrence-free survival for cN0M0 PTC patients

Variable	*p* value	Hazard ratio (95%CI)
Male gender	0.9405	1.04 (0.43–2.46)
Age ≥ 55 years	0.0064	2.23 (1.25–4.00)
Extrathyroid extension (Ex)	0.0002	3.23 (1.74–6.00)
Tumor size		
>4.0 cm	<0.0001	5.68 (2.46–13.16)
2.1–4.0 cm	0.0002	3.70 (1.82–7.14)

**Table 3:** Multivariate analysis of variables, including pathological central node metastasis, for central node recurrence-free survival for cN0M0 PTC patients

Variable	*p* value	Hazard ratio (95%CI)
Male gender	0.9878	0.99 (0.42–2.36
Age ≥ 55 years	0.0028	2.43 (1.36–4.37)
Extrathyroid extension (Ex)	0.0003	3.14 (1.70–5.81)
Tumor size		
>4.0 cm	0.0001	5.10 (2.20–11.90)
2.1–4.0 cm	0.0006	3.34 (1.69–6.67)
Pathologically confirmed	0.0816	1.80 (0.93–3.48)
central node metastasis

**Table 4:** Multivariate analysis of variables, including the number of pathological central node metastases, for central node recurrence-free survival for cN0M0 PTC patients

Variable	*p* value	Hazard ratio (95%CI)
Male gender	0.9446	1.03 (0.43–2.45)
Age ≥ 55 years	0.001	2.60 (1.44–4.69)
Extrathyroid extension (Ex)	0.0002	3.27 (1.76–6.06)
Tumor size		
>4.0 cm	0.0007	4.42 (1.87–10.41)
2.1–4.0 cm	0.0014	3.16 (1.59–6.28)
Pathological central node metastasis ≥5	0.0014	2.65 (1.45–4.83)

### Subset analysis of patients who underwent total, near total, and subtotal thyroidectomy

We performed a subset analysis of patients who underwent total, near total, or subtotal thyroidectomy. These patients underwent also complete p-CND. As shown in Table 5, age ≥55 years, Ex, and large tumor size but not pathologically confirmed central node metastasis independently predicted recurrence to the central compartment. We then performed a multivariate analysis including ≥5 pathological central node metastases instead of pathologically confirmed central node metastasis (Table 6), and we found that ≥5 pathological central node metastasis was also an independent predictor of recurrence to the central node. Thus, the results of our subset analysis are very similar to those obtained for the entire series.

**Table 5:** Multivariate analysis of variables, including pathological central node metastasis, for central node recurrence-free survival for cN0M0 PTC patients, who underwent total, near total, or subtotal thyroidectomy

Variable	*p* value	Hazard ratio (95%CI)
Male gender	0.8297	0.89 (0.31–2.57)
Age ≥ 55 years	0.0033	2.87 (1.42–5.81)
Extrathyroid extension (Ex)	0.0011	3.18 (1.58–6.41)
Tumor size		
>4.0 cm	0.0006	5.29 (2.03–13.70)
2.1–4.0 cm	0.0121	2.89 (1.26–6.67)
Pathologically confirmed central node metastasis	0.3816	1.39 (0.66–2.92)

**Table 6:** Multivariate analysis of variables, including the number of pathological central node metastasis, for central node recurrence-free survival for cN0M0 PTC patients, who underwent total, near total, or subtotal thyroidectomy

Variable	*p* value	Hazard ratio (95%CI)
Male gender	0.8166	0.88 (0.31–2.55)
Age ≥ 55 years	0.0013	3.21 (1.57–6.49)
Extrathyroid extension (Ex)	0.0009	3.28 (1.63–6.62)
Tumor size		
>4.0 cm	0.0031	4.33 (1.64–11.49)
2.1–4.0 cm	0.0212	2.66 (1.16–6.10)
Pathological central node metastasis ≥5	0.0023	2.86 (1.45–5.62)

### Recurrence to the lateral compartment

In our series, 3205 patients underwent p-MND (at least levels II–IV), and p-MND was revealed to have been performed significantly more frequently in patients < 55 years old (*p* < 0.0001), those with a large tumor (cutoffs at 2 and 4 cm, *p* < 0.0001) and those with a tumor having significant extrathyroid extension (*p* < 0.0001). To date, 175 patients (4.1%) in total showed recurrence to lateral compartments.

### Recurrence to distant organs and carcinoma death

Sixty-four patients (1.4%) showed distant recurrence and 39 (0.9%) died of PTC.

### Complications of Surgery

In our series, 237 patients (5.5%) showed permanent hypoparathyroidism. One hundred and seventy-four patients (4.0%) showed permanent vocal cord paralysis, which was due to accidental recurrent laryngeal nerve or vagal nerve injury in 11 patients (0.2%) and due to PTC invasion in the other 163 patients (3.8%).

## Discussion

In this retrospective study, we examined the significance of p-CND for cN0M0 PTC in a large number of patients. Our analyses revealed the following three main findings: (1) pathologically confirmed central node metastasis was significantly linked to aggressive features such as young age, male gender, Ex, and large tumor size; (2) only 1.2% of the patients showed recurrence to the central compartment, and the central node recurrence-free survival rate was excellent at 10 years (99.1%) and 20 years (98.2%); and (3) in a multivariate analysis, old age, Ex, large tumor size and large number of pathological central node metastases (but not the presence of pathological node metastasis) were independent predictors of recurrence to a central node.

The incidence of pathological central node metastasis was higher in the younger patients and in the patients with aggressive clinicopathological features of PTC. The incidence increased significantly with tumor size. Of the PTCs ≤ 1 cm, 43% were positive for pathological central node metastasis, but the incidence rose to 77% in the PTCs > 4 cm (Table 1), which is not discrepant with the results of lateral node metastasis in our previous study [7].

At our hospital in Kobe, Japan, we routinely performed at least ipsilateral CND for PTC patients who underwent a hemithyroidectomy and a complete CND for PTC patients who underwent a total, near total, or subtotal thyroidectomy, similar to many Japanese institutions. In this situation, central node recurrence was not a common event for N0 PTC, because only 1.2% of the present series of patients showed central node recurrence; moreover, the central node recurrence-free survival rate is excellent at 98.2% after a 20-year follow-up. Our multivariate analysis indicates that older age (≥55 years), Ex, and large tumor size independently predicted central node recurrence. It is also worth noting that the incidence of pathologically confirmed central node metastasis was higher in the younger patients but the central node recurrence rate was higher in the older patients. These findings are reasonable because all of the above-mentioned factors were confirmed to be prominent prognostic factors for carcinoma recurrence and carcinoma death [1]. Regarding pathological central node metastasis, our present findings indicated that its presence did not have an independent prognostic impact, but a large number of metastases (≥5) did. Therefore, careful postoperative follow-up is needed for cases that exhibit a large number of central node metastases on pathological examination.

Zhao et al. published a meta-analysis demonstrating that the risk ratio of p-CND for loco-regional recurrence was 0.66, and the number needed to treat (NNT) was 43 [8]. In another meta-analysis, Ma et al. [9] also showed that general risk factors such as old age, male gender, and large tumor size were related to central node recurrence, and they proposed p-CND for patients with these risk factors. One prospective randomized study from Italy showed that p-CND did not improve patients’ prognoses but contributed to a reduced necessity to repeat adjuvant RAI therapy [10]. However, in that study, the number of patients was small at 181 and the mean follow-up time was short at 59.4 months, and all of the patients had undergone adjuvant RAI therapy (at least 30 mCi).

In 2016, Nixon et al. showed that in their series, RAI treatment was performed more frequently for cN0M0 patients with aggressive features such as high T category and high risk based on the GAMES risk stratification system (from the Memorial Sloan-Kettering Cancer Center) and on the ATA risk classification, and therefore, patients without RAI treatment had an even better recurrence-free survival than those with RAI treatment [11]. For their series, at a median follow-up of 46 months, the 5-year disease-specific survival rate was 100%. The 5-year recurrence-free survival and 5-year central neck recurrence-free survival rates were excellent at 96.6 and 99.1%, respectively. Similar to our study, extrathyroid extension independently predicted regional recurrence. Therefore, even though p-CND is omitted for cN0M0 PTC cases without high-risk features such as extrathyroid extension and large tumor size (>4 cm), the central node recurrence-free survival rates might not be significantly changed.

Zao et al. [8] also studied the complications of p-CND in a meta-analysis. They demonstrated that the odds ratios (ORs) of temporary and permanent hypocalcemia in patients who underwent p-CND were high at 2.37 and 1.93, respectively, compared with patients who did not undergo p-CND. Those authors also noted that the ORs of temporary and permanent recurrent laryngeal nerve injury were low at 1.22 and 1.17, respectively, indicating that p-CND increases the risk especially of permanent hypoparathyroidism. At our institutions, permanent hypoparathyroidism occurred in 5.5% of the patients, which may be because we performed a complete p-CND to do total thyroidectomy. If we omit p-CND, this incidence would significantly decrease. In contrast, the incidence of recurrent laryngeal nerve injury at our institutions was very low at 0.2%, even though we performed at least ipsilateral CND; this does not conflict with the results of the meta-analysis by Zao et al.

There are pros and cons of p-CND for cN0M0 PTC patients. If it is not performed, the incidences of permanent hypoparathyroidism would decrease. However, the appearance of central node recurrence during postoperative follow-up is difficult to evaluate, because ultrasound can less clearly show the status of central nodes compared to that of lateral nodes and, unlike lateral nodes, an FNAB for central nodes presents the risk of injuring the recurrent laryngeal nerve. In addition, re-operation for central node recurrence is difficult because it can induce recurrent laryngeal nerve injury and permanent hypoparathyroidism. Another issue is that pN1a is included in TNM staging, and it reflects patients’ prognosis to some extent. However, this information would be lacking if a p-CND is not performed. These factors should be carefully considered when considering whether to perform a p-CND for PTC patients.

If we do not perform routine p-CND, the intra-operative findings must be examined extensively to minimize the recurrence to central nodes. Aspects of central node metastasis such as bulbous appearance and black color can be evaluated to some extent intra-operatively. In addition, a frozen section diagnosis for suspicious nodes might be useful, although this cannot be performed at all institutions or for all cases.

In conclusion, under the situation of routine p-CND, the central node recurrence-free survival of cN0M0 PTC is excellent at 10 (99.1%) and 20 (98.2%) years postoperatively. However, future studies, including double-arm studies from Japan, should examine whether the omission of p-CND cN0M0 PTC is appropriate in consideration of various factors, including the pros and cons of p-CND.
